# Identification of proteins potentially involved in the formation of Lafora bodies, a hallmark of Lafora disease

**DOI:** 10.1186/1750-1326-8-S1-P36

**Published:** 2013-09-13

**Authors:** Elham Schokraie, Oliver Kötting, Matthew S Gentry

**Affiliations:** 1 Department of Biology, Plant Biochemistry, ETH Zurich, Zurich, Switzerland; 2 Department of Molecular and Cellular Biochemistry and Center for Structural Biology, College of Medicine, University of Kentucky, Lexington, Kentucky, USA

## Background

Lafora Disease (LD) is a fatal teenage-onset progressive myoclonus epilepsy. It is characterized by the formation of Lafora bodies (LBs), deposits of abnormally branched, insoluble, hyperphosphorylated glycogen-like polymers that are generally believed to trigger the development of the clinical symptoms of LD. 58% and 35% of the LD cases are caused by mutations in *EPM2A* (laforin) and *EPM2B* (malin), respectively. However, little is known about their function in LB formation. Two different mechanisms have been proposed to explain the accumulation of insoluble LBs: first, excessive glycogen phosphorylation and, second, an imbalance between glycogen synthesizing enzymes. The present study aims at the identification of proteins involved in the molecular mechanisms leading to LB formation and appearance of LD and the phosphorylation of glycogen.

## Materials and methods

Isolation of native LBs is a prerequisite for proteomic analysis of proteins associated with LBs. Therefore, a workflow has been established for the isolation of native LBs from different tissues. Here, we show the analysis of brain, heart and skeletal muscle tissue from 9-month-old *EPM2A*^-/-^ mice. Successful purification of the inclusion bodies was demonstrated by light microscopy after PAS and iodine staining and by electron microscopy. Peptides for analysis on a LTQ Orbitrap Velos were generated by In-solution tryptic digestion of proteins attached to the isolated bodies. To allow for a semi-quantitative analysis, wild-type tissues were prepared in parallel and used as controls. Each experiment was performed with two biological and two technical replicates.

## Results

Using the established workflow we isolated native LBs of variable sizes and shapes from brain, heart and skeletal muscle of *EPM2A*^-/-^ mice. The semi-quantitative analysis resulted in the identification of 90 proteins that were highly upregulated in knockout tissues or not detectable in the wild type. Many of these are well-known proteins involved in glycogen metabolism indicating the significance of our study. However, we also identified a number of interesting candidates potentially or de facto involved in disease related processes, such as autophagy.

## Conclusions

We established the first workflow for the isolation of native LBs allowing for a subsequent proteomic analysis of associated proteins. Using this method, we identified known glycogen-related proteins together with new candidates putatively involved in LB formation and LD establishment representing potential targets for future therapeutic approaches.

